# Exploring perspectives of Scottish medical students on the proposed ‘Assisted Dying for Terminally Ill Adults (Scotland)’ Bill

**DOI:** 10.1186/s12910-025-01322-1

**Published:** 2025-12-11

**Authors:** David Geddes, Josh Knox, David Obree, Jeni Harden

**Affiliations:** 1 Edinburgh Medical School, University of Edinburgh, Edinburgh, United Kingdom; 2 Usher Institute, University of Edinburgh, Edinburgh, United Kingdom

**Keywords:** Ethics, Medical, Bioethics, Personal Autonomy, Suicide, Assisted, Right to Die, Terminal Care, Palliative Care, Legislation, Medical, Students, Medical, Legislative Ethics

## Abstract

**Background:**

The debate over legalising assisted dying is complex and multifaceted, eliciting strong and diverse opinions from all involved parties. Recently, the Scottish Parliament held an initial vote on the issue, opting to progress with proposed legislation. Hearing the perspectives of medical students, as future clinicians, is crucial when considering new medico-legal developments in assisted dying in Scotland. This study aimed to explore and examine medical students’ views on assisted dying in Scotland and the specific provisions of the proposed Scottish legislation.

**Methods:**

An online survey was distributed to students at the Scottish medical schools between October 2023 and January 2024. Participants were invited to respond to closed, quantitative questions, designed to assess specific attitudes towards the proposed legislation, followed by optional free-text fields for participants to provide qualitative explanations for their answers. These free-text responses were subsequently subjected to thematic analysis.

**Results:**

A total of 295 students responded to the survey. The majority of respondents were in favour of the legalisation of assisted dying in Scotland (72.4%); however, only 48.5% of respondents thought that assisted dying should be introduced into Scottish legislation as proposed in the current bill. Additionally, 23.4% of respondents indicated they would conscientiously object to participation in the process. A significant number of respondents (*n* = 135) expressed concerns that the proposed safeguards were inappropriate or insufficient. Autonomy was the most cited medical ethics principle underpinning opinions, followed closely by non-maleficence.

**Conclusions:**

The majority of respondents were in support of assisted dying, but a smaller proportion felt the current proposed bill should be implemented, due to different interpretations of the suitability of proposed safeguards. A significant portion of respondents’ perspectives were based on information from their previous training and experience within healthcare. Other perspectives mirrored wider public opinion on end-of-life care and assisted dying principles. While a majority of respondents favour legalising assisted dying in Scotland, significant concerns remain regarding the specific provisions of the proposed bill, highlighting the complex and ethically charged nature of this issue. Key stakeholders and policymakers should actively involve medical students, as future medical practitioners, in the assisted dying discussion to help guide legislation and research.

**Supplementary Information:**

The online version contains supplementary material available at 10.1186/s12910-025-01322-1.

## Introduction

Assisted dying refers to the process of medical practitioners “*prescribing life-ending drugs for terminally ill*,* mentally competent adults to administer themselves after meeting strict legal safeguards*” [1]. Over recent years, as international legislation has been introduced, assisted dying has become an increasingly discussed and debated topic, often eliciting strong and opposing views [2].

The debate surrounding assisted dying is complex and multifaceted, intersecting medical law, medical ethics, and religious thought [2–4]. Arguments in favour of legalisation centre on two intersecting principles: personal autonomy and relief of suffering. Proponents make the point that allowing a mentally competent adult, when facing a terminal illness, to choose the manner and timing of their death is a fundamental expression of their self-determination. They posit that legal access to assisted dying is not just about ending suffering but empowering an individual to make a truly autonomous choice when faced with intractable illnesses and intolerable pain. Even where high-quality palliative care is available, it is argued that not all individuals will achieve adequate relief from their suffering and pain, and assisted deaths should remain an option for those for whom suffering persists.

Conversely, opponents of assisted dying argue that legalisation could give rise to a range of adverse consequences, including the devaluation of life, erosion of trust in the medical profession, and heightened risk to vulnerable groups. The concept of the ‘sanctity of life’ is frequently cited, with critics from both secular and religious backgrounds maintaining that the intentional ending of life fundamentally breaches this core moral value [5]. Another concern of those against legalisation is the so-called ‘Slippery Slope’ argument, wherein the scope of assisted dying may gradually expand beyond its original intent [6]. Additionally, there is significant apprehension regarding the potential impact of such legislation on vulnerable groups, including those of lower socioeconomic status, those with disabilities, and those lacking decision-making capacity [5].

In recent decades, discussions surrounding end-of-life choices in Scotland have mirrored a growing international trend, with countries such as Australia, Belgium, Canada, and the Netherlands also at the forefront of legislative reform [7, 8]. In March 2024, a bill seeking to legalise assisted dying for terminally ill adults was introduced into the Scottish Parliament after previous attempts in 2010 and 2013 were unsuccessful [9]. The definition of assisted dying used in the Scottish legislation aligns closely with other legislative frameworks, including the English *‘Terminally Ill Adults (End of Life) Bill’* [10]. Core principles of these frameworks include a diagnosed terminal illness and the capacity for decision-making. A summary of the key aspects of the Scottish legislation is shown in Table 1.

**Table 1:** Shows a summary of the key aspects of the proposed “Assisted Dying for Terminally Ill Adults (Scotland)’ Bill

1. Eligibility Criteria
The legislation requires the individual to be a Scottish resident, aged 16 or over, with a diagnosed terminal illness and decision-making capacity
2. Request & Assessment Process
Legislation requires two formal patient declarations and two independent medical practitioners to confirm all eligibility and safeguarding criteria have been met
3. Key Safeguards
Legislation includes a 14-day reflection period, an explicit right for the person to withdraw at any time, and makes coercion a criminal offence
4. Method of Assistance
Legislation limits the method of assistance strictly to self-administration of an approved substance provided by a healthcare professional, who must remain present with the person
5. Conscientious Objection
The legislation allows any individual the explicit right to refuse to participate in the process, removing any legal or professional duty to act

In Scotland, students enrolled in medical degree programmes (MBChB or equivalent) are in the process of training to become qualified doctors. As the future generation of clinicians who, if legislation passes, would be critically involved in the assisted dying process, medical students’ perspectives are essential when considering new medico-legal developments such as assisted dying. Whilst several professional medical organisations – including BMA Scotland and the Royal College of Physicians of Edinburgh – have been consulted regarding the proposed legislation, there have been no formal efforts by the Scottish Parliament to engage with representative bodies for medical students during the legislative process.

To address this gap, we are the first to report the views and perspectives of medical students across the five Scottish Universities offering MBChB or equivalent degrees. We investigated medical students’ general opinions on assisted dying, as well as their responses to the specific provisions of the current bill. Importantly, we highlight the collective viewpoints of future clinicians, as well as offering future recommendations for legislators and clinicians.

## Methods

### Survey design

An Online Survey was developed using the Jisc OnlineSurveys V2.0 tool. The online survey comprised a combination of closed questions, with participants able to provide free-text responses for each. In addition to collecting basic demographics, the survey included questions designed to assess participants' perspectives on assisted dying and specific aspects of the proposed “*Assisted Dying for Terminally Ill Adults (Scotland) Bill*”- hereafter referred to as the “proposed bill” (Table 2). These questions addressed: overall attitudes towards assisted dying, whether the bill should be implemented as currently drafted, the possibility for conscientious objection, the quality of the proposed safeguards, and potential impacts on vulnerable people. For questions that required participants to understand the proposed Scottish legislation, the relevant information was provided throughout the survey (Supplementary Material 1).

**Table 2:** Shows the questions included in the survey

1. What is your stance on having Assisted Dying as a legal option for terminally ill adults in Scotland?
2. Should the ’Assisted Dying for Terminally Ill Adults (Scotland) Bill’ be implemented as it currently stands?
3. If the bill were to be become legislation, would you (as a future practitioner) conscientiously object?
4. If the bill were to be implemented as it stands, which of the following best characterises your viewpoint on the proposed safeguard?
5. How do you think this bill will impact vulnerable people e.g. those at risk of abuse or neglect due to the actions (or lack of action) of another person?
6. If you have further comments on the bill, or on the topic of Assisted Dying in Scotland, please provide them here:

*This* *does not include the basic demographic questions which can be found in supplementary material*

### Survey distribution

The survey was distributed to the five Scottish Universities offering an MBChB (or progression towards equivalent) degree as follows: The University of Aberdeen, The University of Dundee, The University of Edinburgh, The University of Glasgow, and The University of St Andrews.

The survey was distributed and open between October 2023 and January 2024. Distribution was conducted through internal university channels, including cohort-wide emails, advertisements during lectures, and posters displayed within medical school facilities. Therefore, the precise number of students who were invited to complete the survey could not be determined. During the 2023/2024 academic year, there was a total of 7,135 enrolled medical students across all Scottish medical schools [11]. Based on this total student population, the 295 responses collected for this study represent an approximate total response rate of 4.1%.

### Data analysis

Closed answer, quantitative data was exported and analysed using Microsoft Excel. Duplicate or blank free-text responses were excluded from analysis. Participants were not required to provide free-text responses to each question to progress to the next question.

Free-text responses were exported to Microsoft Excel and subsequently transferred to NVIVO (Lumivero) for qualitative analysis. First, responses to each free-text question were analysed independently using an inductive approach, building exploratory themes in a bottom-up approach where the researchers generated initial codes and themes that emerged directly from the content of the responses [12]. A single response could be assigned multiple codes if it contained several distinct ideas. Following this, a deductive (top-down) approach was undertaken. All the free-text responses were collectively analysed against a predefined framework of the four pillars of medical ethics: autonomy, non-maleficence, beneficence, and justice. Quantification of each theme was then undertaken to compare the prevalence of particular viewpoints among respondents (*n* = 771) [13].

### Ethical considerations

Given the sensitive nature of the topic of assisted dying, special care was given to the design, implementation, and analysis of the study and its data. The study was reviewed and fully approved by the University of Edinburgh Medical Education Research Ethics Committee. During survey creation, careful consideration was given to the content of the questions, to minimise risk for distress and to maintain neutrality. Participants were explicitly informed they were free to withdraw from the study at any stage and were provided the option to report any complaints to the authors.

## Results

### Demographics

A total of 296 responses were received, with one duplicate removed from analysis and quantification, giving a total number of 295 responses. Specific demographic data is shown in Table 3.

**Table 3:** Highlights summary data of all respondents, n = 295. Percentages may not sum to 100 due to rounding

Demographic		Number Of Respondents	% of respondents
University	University of Aberdeen	164	55.6
	University of Edinburgh	62	21.0
	University of Dundee	41	13.9
	University of Glasgow	20	6.8
	University of St. Andrews	8	2.7
	**Total**	**295**	**100.0**
Year Group	First Year	101	34.4
	Second Year	44	15.0
	Third Year	59	20.1
	Fourth Year	50	17.0
	Fifth Year	35	11.9
	Sixth Year	5	1.7
	**Total**	**294**	**100.0**
Age	Under 18	8	2.7
	18–21	163	55.3
	22–25	79	26.8
	Above 25	45	15.3
	**Total**	**295**	**100.0**

### Assisted dying in Scotland

When asked whether assisted dying should be a legal option for adults in Scotland with a terminal illness, 213 respondents (72.4%) were in favour, 59 (20.1%) were against, and 22 (7.5%) were unsure or had no opinion (Table 4 A).

**Table 4:** Summarises respondents’ views on assisted dying in Scotland. A. Highlights views on legalising assisted dying in principle. B. shows views on implementing the bill in its current form

A. What is your stance on having assisted dying as a legal option for terminally ill adults in Scotland?		
Response	**N. of Respondents (*n* = 294)**	**%**
Yes	213	72.4
No	59	20.1
Unsure/No Opinion	22	7.5

B. Should the ‘Assisted Dying for Terminally Ill Adults (Scotland) bill’ be implemented as it currently stands?		
Response	**N. of Respondents (*n* = 295)**	**%**
Yes	143	48.5
No	96	32.5
Unsure/No Opinion	56	19.0

Among those in favour, the most cited justification was upholding and promoting patient autonomy (*n* = 97, 63.8%), followed by the prevention of harm and suffering (*n* = 92, 60.5%). Additional reasons included maintaining dignity (*n* = 38, 25.0%), improving quality of life (*n* = 13, 8.5%), or reducing harm to loved ones (*n* = 9, 5.9%).

Among respondents who were opposed, primary concerns pertained to the principle of non-maleficence (causing direct harm) (*n* = 21, 43.8%) and the risk of coercion (*n* = 21, 43.8%). Other objections were due to respondents’ religious beliefs (*n* = 19, 39.6%), concerns about normalising suicide (*n* = 11, 22.9%), fears of slowing research into curative treatments (*n* = 6, 12.5%) and the risk of financial factors influencing decisions (*n* = 3, 6.25%).

“*To choose the manner of one’s death, in a comfortable environment, with loved ones. should be the right of individuals with a terminal diagnosis when the alternative is to lose themselves in pain.” Response 109, University of Aberdeen, Year 5, Above 25y*

“*We should focus on helping patients get better rather than finding a “simple” solution to kill.” Response 81, University of Dundee, Year 3, Above 25y*

When asked whether the proposed bill should be introduced in its current form, 143 respondents (48.5%) were in favour, 96 (32.5%) were opposed, and 56 (19.0%) were unsure or had no opinion (*Table 4B*).

Of the free text responses received to this question which supported the bill (*n* = 36), the primary justification involved maintaining personal autonomy (*n* = 13, 36.1%) and reducing suffering (*n* = 13, 36.1%). However, the majority of free text responses received opposed the bill in its current form (*n* = 65), raising concerns about provisions in the proposed bill. The most common concern was the proposed minimum age limit of 16 (*n* = 21, 32.3%), followed by risks of misinterpretation (*n* = 15, 23.1%), its restriction to those with a terminal illness (*n* = 11, 17.0%) and the bill’s definition of ‘terminal illness’ (n = 9, 13.8%).

“*It gives them a choice, a choice their illness might have deprived them of, a choice to manage their own treatment and decide whether they want to keep going or not.” Response 288, University of St. Andrews, Year 1 18y.*

*“I think the complexities of human life and the uncertainty of the future would put me in a position as a doctor where I would feel at great discomfort to say that a certain medical condition will likely cause death.“ Response 41, University of Aberdeen, Year 3, 25y.*

### Conscientious objection

In answer to the question, would you (as a future practitioner) conscientiously object if the proposed bill became legislation, 69 respondents (23.4%) selected the option that they would conscientiously object, 182 (61.7%) selected they would not, and 44 (14.9%) selected unsure/no opinion (Table 5).

**Table 5:** Summarises respondents’ views on conscientious objection to assisted dying in Scotland

If the bill were to become legislation, would you (as a future practitioner) conscientiously object?		
Response	**N. of Responde** **nts (** ***n*** ** = 295)**	**%**
Would	69	23.4
Would Not	182	61.7
Unsure	44	14.9

Of the free-text responses received from those who would conscientiously object (*n* = 38), the most cited rationale was conflict with personal morals or beliefs, which respondents attributed to religious beliefs (*n* = 14, 36.8%) or the notion that being involved in such a procedure would be doing direct harm (*n* = 15, 39.5%).

Of those free text responses from those who would not conscientiously object (*n* = 63), many indicated their willingness to participate was to support patient autonomy (*n* = 28, 44.4%) and minimise harm or suffering of terminally ill adults (*n* = 23, 36.5%). A further subset viewed participation as an integral professional duty or ‘*part of the job*’ (*n* = 12, 19.0%).

*I want more families to have a peaceful goodbye to their loved ones which allows them to remember them how they wish to be remembered and would be happy to provide people with that opportunity*.*Response 26*,* University of Aberdeen*,* Year 3*,* 20y.*

*“Morally*,* my job as a practitioner is to make life better for patients not to take it away.“ Response 40*,* University of Aberdeen*,* Year 1*,* 20y.*

### Safeguards

Respondents expressed nuanced opinions on the bills’ proposed safeguards, with a clear majority (84.3%) finding them appropriate in principle but divided on their sufficiency. When asked which statement best characterised their viewpoint of the bill’s proposed safeguards, 145 respondents (49.5%) chose “appropriate and sufficient”, 102 (34.8%) chose “appropriate but insufficient”, 33 (11.3%) chose “inappropriate and insufficient”, and 13 (4.4%) chose “Other” (Table 6).

**Table 6:** Highlights respondents’ responses on the appropriateness/sufficiency of the bill’s proposed safeguards

If the bill were to be implemented as it stands, which of the following options best characterises your viewpoint on the proposed safeguards?		
Response	**N. of Respondents (** ***N*** ** = 293)**	**%**
Appropriate and Sufficient	145	49.5
Appropriate but Insufficient	102	34.8
Inappropriate and Insufficient	33	11.3
Other	13	4.4

Of the written responses received (*n* = 113), multiple concerns were shared across each subset of respondents, with some feeling that the safeguards were too strict, whilst others felt they were too loose. The primary overarching issue related to the requirement for self-administration of the life-ending medications, with concerns that this may disproportionately disadvantage those lacking the physical capabilities to do this (*n* = 25, 22.1%). Another shared concern was the specific healthcare practitioners who were to be involved in the assisted dying process, for example, the need for specialist assisted dying or psychiatric medical staff or associated staff such as social workers (*n* = 22, 19.5%).

Of the respondents who felt the safeguards were either “appropriate but insufficient” or “inappropriate and insufficient,” two primary concerns were shared (*n* = 58). Firstly, 18 respondents felt that the proposed safeguards did not do enough to protect vulnerable people from coercion, arguing that in the bill’s current form, vulnerable groups may be pressured into seeking assisted dying for inappropriate reasons, e.g. financial or social (*n* = 18, 31.0%). Secondly, respondents raised concerns about the proposed 14-day reflection period of the bill (i.e. the minimum time period after which a patient signs their written declaration before they are eligible to receive the life-ending medications). These concerns were conflicting, however, with 10 respondents (*n* = 10, 17.2%) feeling that 14 days was too short and 3 (*n* = 3, 5.2%) stating it was too long. Respondents who considered 14 days too short felt that it would not be enough time to make such an important decision, whilst those who felt that it was too long and would negatively impact those with prognoses shorter than 14 days.

Further concerns from these respondents highlighted the eligibility of those lacking capacity (*n* = 4, 6.9%), for example, those with cognitive impairment or those in mental distress, whilst other concerns were raised about the potential risk for human error at any stage in the process (*n* = 3, 5.2%).

### Overarching pillars

In addition to the inductive analysis, a deductive analysis was performed to quantify the use of foundational ethical principles in students’ reasoning. Deductive analysis of the free text responses revealed that participants’ reasoning was frequently underpinned by one or more of the four pillars of medical ethics, despite the survey containing no prompts to do so [13]. Application of these principles without prompting highlights the approach of respondents when faced with this bioethical issue. Across all free-text responses, a total of 771 instances of this ethical reasoning were coded. Using the established definitions, responses were thematically coded according to the pillar that best reflected the participant’s reasoning.

Autonomy emerged as the most frequently cited ethical principle (*n* = 283, 36.7%), predominantly among those in favour of assisted dying. These respondents typically framed autonomy as the ability to maintain personal control over end-of-life decisions. Following this, non-maleficence was the second most cited pillar (*n* = 259, 33.6%), more commonly referenced by those opposed to assisted dying, who felt that either the act itself constituted direct harm or that the proposed safeguards were insufficient to prevent such harm.

A key finding that illustrates the complexity of the debate is that foundational principles, including beneficence and justice, were used to justify entirely opposing views. The principle of beneficence was cited by both supporters and opponents of assisted dying (*n* = 159, 20.6%), though with differing interpretations. Those in favour associated beneficence with prioritising patients’ comfort, dignity, and quality of life over the mere prolongation of life. In contrast, opponents appealed to beneficence in arguing that individuals may feel pressured or coerced into seeking assisted dying, which they believed would not be in the individuals’ best interests. This finding suggests that this complex debate is not about underpinning ethical principles themselves, but rather their interpretation.

Finally, the pillar of justice was also put forward as an argument by both groups (*n* = 70, 9.1%), particularly in relation to the safeguards outlined in the proposed legislation. The most prominent concern related to justice involved the requirement for participant self-administration. Respondents questioned the fairness of access to assisted dying for those who may lack the physical ability to self-administer medications.

## Discussion

### Assisted dying in Scotland

The majority of respondents (72.4%) supported legalising assisted dying for adults with a terminal illness, aligning with previous public surveys in Scotland (70–75%) [14, 15]. Free-text responses highlighted that the primary justifications amongst those in favour relate to personal autonomy and alleviation of suffering. These justifications, particularly the emphasis on autonomy and the relief of suffering, reflect not only key principles in the field of bioethics but also a developing international legal consensus grounded in human rights law, including rulings from both the European Court of Human Rights and the United Nations Human Rights Committee [16–20]. The emphasis on dignity, quality of life, and distress further reflects widely recognised principles in the field of palliative care and medical ethics [21].

*“People can choose how they want to live*,* so why can’t they choose if they want to die?” Response 34*,* University of Aberdeen*,* Year 1*,* Above 25y*.

*“Terminally Ill Adults… suffer… just for them to face death at the end. No one should be going through that. I believe people should have a choice in the matter – A choice to not go through that.” Response 19*,* University of Aberdeen*,* Year 3*,* 21y*.

*“Leaving people to suffer the harshness of the world is not what modern medicine has evolved for.” Response 48*,* University of Aberdeen*,* Year 1*,* 22y*.

Despite majority support, 20.1% of respondents opposed introducing assisted dying, with major concerns centred on causing direct harm, the risk of coercion and religious/moral viewpoints. These concerns mirror broader opposition to assisted dying, particularly concerns that legalisation of assisted dying might cause direct harm, as well as opening risks of coercion to seek assisted dying [22]. The reasons for objection cited in this study are deeply intertwined with the respondents’ medical training, reflecting broader debates around the role of physicians in end-of-life care and their professional and moral duties [23, 24].

*“As I understand it*,* doctors are in the business of healing people*,* not killing*,* however you wish to frame it. Have we become so proud and godless to think we hold the keys to life and death?” Response 44*,* University of Aberdeen*,* Year 4*,* 25y*.

*“I believe in the sanctity of life. I believe my role as a future doctor will be to preserve life and not to play God. I would sooner forfeit my career than play any part in consciously and intentionally ending the life of another human.” Response 172*,* University of Aberdeen*,* Year 5*,* 25y*.

Interestingly, support for the current form of the “Assisted Dying for Terminally Ill Adults (Scotland) Bill” was lower than general support for legalisation, suggesting differing views on the bill’s specific provisions (72.4% vs. 48.5%). The most frequently cited concern was the proposed minimum age of sixteen, with respondents questioning whether individuals at this age possess the requisite capacity to make such a profound decision. This concern reflects broader medico-legal and ethical discussions around the ability for younger people to make decisions pertaining to their end-of-life care [25–27].

Primary protections for vulnerable groups in the proposed bill include a 14-day reflection period, an explicit right for the person to withdraw at any time and the criminalisation of coercion. However, major concerns were raised about the possible risk of misinterpretation or abuse of the bill, with respondents questioning whether these current protections for vulnerable groups were sufficient. Opposition to assisted dying often highlights risks posed by family members, caregivers, financial pressures, and healthcare systems, which may influence individuals’ decisions [22].

The level of support for assisted dying in principle amongst medical students (72.4%) was notably higher than that reported among practising UK physicians, where the most recent large-scale surveys typically showed support of around 40–50% [28, 29]. Furthermore, the medical students’ majority support contrasts with the official position of neutrality adopted by key professional bodies, including the Royal College of Physicians of Edinburgh and BMA Scotland [30, 31]. This discrepancy suggests a potential generational shift, which could be attributed to factors such as evolving societal opinions regarding patient autonomy and differences in medical ethics training [17]. However, it may also reflect a distinction between the more theoretical viewpoint of medical students versus the viewpoint of clinicians currently working in healthcare [32].

### Conscientious objection

Interestingly, there was a notable difference between the level of absolute support for legalisation of assisted dying (72.4%) and the proportion of respondents who would be definitely willing to participate in the process (61.7%). This may suggest a potential gap between supporting the principle and a personal willingness to enact it, although this interpretation may be impacted by the portion who were ‘unsure’ if they would conscientiously object (14.9%). Conscientious objection in end-of-life decisions is crucial, allowing practitioners to exert their own personal autonomy in their involvement. Analysis of free-text responses highlighted the main motivations for conscientious objection as being religious or moral beliefs, findings seen in previous literature [33–35]. If implemented, it would be vital to include principles and clauses for those who wish to conscientiously object, whilst ensuring patients continue to receive fair access to care.

*“Just as I would never help a patient push a knife deeper into their chest*,* I could never give a patient drugs with which to kill themselves.” Response 44*,* University of Aberdeen*,* Year 4*,* 25y*.

### Safeguards

While a majority of respondents (84.3%) considered the proposed safeguards ‘appropriate’ to some degree (i.e. ‘appropriate and sufficient’ or ‘appropriate but insufficient’), only 49.5% believed them to be fully ‘sufficient’. This indicated that while current safeguards are valuable, additional measures are needed before implementation. The two primary perspectives from those who felt the safeguards were appropriate but insufficient related to self-administration of life-ending medications and the required medical practitioners involved. The concern around self-administration has been seen in other jurisdictions which have legalised assisted dying, with some countries opting to legalise the ability for the clinician to administer the medications [36–38].

*“I feel this discriminates against people with disabilities who are physically unable to administer the medicine themselves… additional measures should be in place to enable disabled persons access to the same standard of healthcare as physically able patients.” Response 57*,* University of Aberdeen*,* Year 1*,* Above 25y*.

Furthermore, respondents felt that a wider range of healthcare professionals, such as psychiatrists, nurses, and social workers, should be involved in the process. Previous research has highlighted a lack of specific definitions of these roles in the process of assisted dying [38–40]. To ensure clarity, it would be beneficial if the Scottish bill were to clearly define the roles and duties of other healthcare professionals involved in the process.

Following this, further major concerns related to the proposed safeguards mirror wider societal debates addressing the risks of coercion within assisted dying. Research from other jurisdictions has highlighted the roles of family members in assisted dying discussions, possibly leading to emotional and pressured environments for decision making, although previous research has been unable to find an increased risk of coercion in vulnerable groups [22, 41–43]. Researchers have proposed specific frameworks to assess the risk of coercion in assisted dying processes, which may offer more strength to the proposed Scottish safeguards [44].

The concerns regarding safeguards raised by medical students in this study align closely with requirements set out by international human rights bodies, which state that assisted dying can be compatible with human rights, provided what the UN Human Rights Committee terms *‘robust legal and institutional safeguards’* are in place [18–20]. These safeguards must be shown to protect vulnerable people, ensure autonomy and voluntary decisions, and require independent monitoring [18–20].

### Limitations and next steps

Whilst this study provides valuable insight into medical students’ attitudes on the topic, it is important to acknowledge some limitations. Firstly, the sample size of the study represents only a small proportion of all Scottish medical students (4.1%) and may reflect a self-selected group with particular interests in the topic. Due to the survey being distributed through internal university channels, the total number invited to participate in the survey is not known. Further studies should aim for broader representation across institutions and year groups to provide a more generalisable understanding.

Furthermore, this study did not collect data on the specific medical education regarding end-of-life care and assisted dying that respondents had received prior to survey completion. Given the likely heterogeneity in curricula across the Scottish medical schools, this variation in education represents a potential confounding variable. Further research should therefore aim to correlate participants’ ethical viewpoints with their level of medical education on this topic.

The disproportionate response rates from different institutions may be attributed to two factors. Firstly, differences in the distribution and advertisement of the survey within each institution may have influenced participation. Secondly, varying levels of interest and engagement in medical ethics fields, including assisted dying, may exist between Scottish medical schools. Future research could explore whether teaching approaches or curriculum differences influence students’ perspectives on assisted dying.

The largest proportion of respondents were in their first year of study (*n* = 101, 34.2%), with participation decreasing among senior year groups. This may reflect differences in engagement with online surveys or the possible increased workload of later-year students. Given that senior students have greater clinical exposure, their perspectives on assisted dying may change over time. Further research should be undertaken to investigate whether attitudes shift as students’ progress through their medical education and training.

Interestingly, the use of the four pillars of medical ethics by respondents – albeit in varied ways and differing frequencies – emerged as an incidental finding, rather than a predefined study objective. This indicates that these ethical principles form a foundational component of medical students’ reasoning around assisted dying, even without explicit prompting. Future research should be designed with a specific focus on exploring how these foundational principles influence students’ perspectives and would offer deeper insight into the ethical frameworks that underlie future clinicians’ positions.

## Conclusion

Assisted dying remains a complex and ethically charged topic, eliciting perspectives that span the full breadth of the medical ethics continuum. The perspectives of Scottish medical students are vital, as these future practitioners will be directly involved in the potential implementation of assisted dying.

This study showed that the majority of medical students surveyed were in support of legalising assisted dying for terminally ill adults in Scotland. Whilst the majority support assisted dying, a smaller majority were supportive of implementing the proposed *‘Assisted Dying for Terminally Ill Adults (Scotland)’* bill. A substantial number of respondents raised concerns about the specifics of the outlined bill, particularly the proposed eligibility criteria, proposed safeguards, and the necessity for rigorous protection against the bill’s abuse.

Moving forward, we propose the following recommendations to ensure the perspectives of medical students are meaningfully integrated into the legislative process:

1. Professional medical organisations, such as BMA Scotland and the Royal Colleges, should create formal mechanisms to include the perspectives of medical students as a cohort when interacting with policymakers and stakeholders during the development and introduction of legislation.
2. Medical schools and student bodies should actively empower students by creating opportunities for them to take a proactive role in discussions around end-of-life care and assisted dying during training.
3. Further research, including longitudinal studies, should be conducted into medical students’ contributions to the assisted dying debate to ensure their contributions meaningfully inform legislative and professional consultations.

We are the first to report Scottish medical students’ perspectives and opinions on assisted dying and the proposed bill. We advocate that policymakers and key stakeholders engage with medical students in ongoing and future consultations.

## Supplementary Information

Supplementary Material 1.

## Data Availability

The datasets used and/or analysed during the current study are available from the corresponding author on reasonable request.
