# Supplementary material for: Exploring perspectives of Scottish medical students on the proposed ‘Assisted Dying for Terminally Ill Adults (Scotland)’ Bill
Source: BMC Med Ethics. 2025 Dec 11;26:173. doi: 10.1186/s12910-025-01322-1 (PMC12699872; doi:10.1186/s12910-025-01322-1)
Supplement: Supplementary file 1 — Supplementary Material 1. [file 12910_2025_1322_MOESM1_ESM.pdf]

Assisted Dying Survey

p. 1 Disclaimer

Add item

Exploring perspectives of Scottish Medical Students on the proposed 'Assisted Dying for Terminally Ill Adults (Scotland)' Bill

Add item

Information about the research

Please take time to read the following information carefully, if you require any further information before deciding to take part or not, please contact us at [redacted] If you are wanting to take part in the survey, but would rather provide verbal responses, please contact us.

What is the purpose of the study

The Terminally Ill Adults Bill is currently going through the Scottish Parliament. You can find information about the Bill on the Scottish Parliament website - https://www.parliament.scot/bills-and-laws/proposals-for-bills/Proposed-Assisted-Dying-for-Terminally-Ill-Adults-Scotland-Bill and a summary of the consultation at https://www.assisteddying.scot/ . The views of medical students were not explicitly sought during the consultation. This project aims to gather Medical Students' opinions and thoughts on assisted dying as a topic, as well as specifics on the Assisted Dying for Terminally Ill Adults (Scotland) Bill.

Who is organising and funding the research

The project is being organised by the lead researchers - [redacted] Medical Students at the University of Edinburgh, in partnership with staff in the College of Medicine and Veterinary Medicine (CMVM) and the Usher Institute (https://www.ed.ac.uk/usher). No funding is being received for the project and future funding needs are not anticipated.

Why have I been asked to take part

You have been asked to take part because you are a Medical Student at one of the 5 Scottish Medical Schools (Aberdeen, Dundee, Edinburgh, Glasgow, St Andrews). The topic of assisted dying can be emotionally challenging, you do not have to take part in the survey all participation is entirely voluntary Consent to participate will be confirmed both at the beginning and end of the survey. All survey responses will be anonymised, so it is not possible to withdraw data after submission.

What will happen if I take part?

The survey will take no more than 20 minutes. The survey will ask a series of questions aiming to gather information/opinions on the following;

1. Assisted dying
2. The bill currently before Scottish Parliament, focussing on
    1. Proportions for/against inception of the bill
    2. General opinions on the bill and its implementation
    3. Perspectives and concerns regarding the current safeguarding proposals, additionally - any areas of worry for abuse of these safeguards, including those affecting minority groups who may encounter negative discrimination through perverse interpretation of the bill.

The questions will be both closed, quantitative data questions aiming to gather statistical data as well as open, free text responses, aiming to hear your opinion on assisted dying as a topic as well as perceptions/opinions of the bill in the Scottish Parliament.

At the start of the survey, we will ask you some demographic questions about your background. You can choose to not answer any of the questions in the survey without giving reason.

What happens to my information

All personal information during the research will be kept entirely confidential and all survey responses will be anonymised and held securely, and unique ID numbers will be assigned to each survey participant on data analysis. All data featured in publications, presentations and reports will not be identifiable and will use the assigned participant IDs. Demographic information gathered may refer to specific groups, but no information that risks an individual being identifiable will be shared. When quotes are used in the data, each will be assigned to 'Participant 1' etc with no further identifiable features.

All ethical and legal guidelines will be followed, and all information will be kept privately and securely. All electronic data will be stored in a password protected data store and all data files will be kept for a maximum of 5 years. If there are any questions regarding how information is held, contact the University of Edinburgh Data Protection Officer [redacted]

What will happen to the results of the research

The results of the survey and study may be published as articles, presentations, reports, or abstracts and may be used by the Scottish Parliament to inform policy. You will be unable to be identified in any of the published results.

Who has reviewed the study

The study has been reviewed and approved by Edinburgh Medical School Medical Education Unit (REC) [redacted]

Complaints

If there are any complaints/concerns about the research, please contact the Usher Institute Director of Education, Dr Jeni Harden [redacted]

p. 2 Consent

Add item

CONSENT

Please read the following statements -

I understand that all participation in the project is voluntary, and I can stop the survey at any time.

I understand that the content of this survey may be emotionally challenging, and I am aware that I can stop at any time.

I understand that all my responses will remain confidential and no identifiable information will be used in the outcomes of the research.

I understand that by completing the survey and clicking finish, an indication of implied consent is taken.

Add item

1 Agreement To Proceed *

Yes

Add item

Add item

p. 3 Demographics

Add item

2 What Medical School do you attend?

- University of Aberdeen
- University of Dundee
- University of Edinburgh
- University of Glasgow
- University of St. Andrews

Show less

Add item

Add item

3 What year are you in?

- Year 1
- Year 2
- Year 3
- Year 4
- Year 5
- Year 6

Show less

Add item

4 What age are you?

- Below 18
- 18
- 19
- 20
- 21
- 22
- 23
- 24
- 25
- Above 25

Show less

Add item

Add item

p. 4 Exploring perspectives of Scottish Medical Students on the proposed 'Assisted Dying for Terminally Ill Adults (Scotland)' Bill

Add item

5 What is your stance on having Assisted Dying as a legal option for terminally Ill Adults in Scotland?

- For
- Against
- Unsure/Don't have an opinion

Add item

a If comfortable to do so, please give the main reasons why you have made the above choice/hold the above view.

Add item

Add item

Add item

p. 5 Exploring perspectives of Scottish Medical Students on the proposed 'Assisted Dying for Terminally Ill Adults (Scotland)' Bill

Add item

The aim of the proposed 'Assisted Dying for Terminally Ill Adults (Scotland)' bill is to enable mentally competent adults who are terminally ill to be provided with assistance to end their life at their request.

In Scotland, a person is terminally ill if a registered medical practitioner has diagnosed them as having a progressive disease, which can reasonably be expected to cause their death.

The person must be 16 years of age or over, which is the age of majority in Scotland, and have been a resident of Scotland for at least twelve months.

Add item

6 Should the 'Assisted Dying for Terminally Ill Adults (Scotland) Bill' be implemented as it currently stands?

- Yes
- No
- Unsure/Don't have an opinion

Add item

a If comfortable to do so, please give the main reasons why you have made the above choice/hold the above view?

Add item

Add item

7 If the bill were to become legislation, would you (as a future practitioner) conscientiously object?

- Yes
- No
- Unsure/Don't have an opinion

Add item

a If comfortable to do so, please give the main reasons why you have made the above choice/hold the above view?

Add item

Add item

Add item

p. 6 Proposed Safeguards

Add item

Proposed Safeguards in the 'Assisted Dying for Terminally Ill Adults (Scotland)' Bill

- *Two doctors independently confirm the person is terminally ill.*
- *Two doctors establish that the person has the mental capacity to request an assisted death.*
- *If either doctor is unsure about the person's capacity to request an assisted death, the person is referred to a psychologist or other appropriate specialist.*
- *Two doctors assess that the person is making an informed decision without pressure or coercion.*
- *Two doctors ensure the person has been fully informed of palliative, hospice, and other care options.*
- *The person signs a written declaration of their request, which is witnessed and signed by both doctors.*
- *A suggested waiting period of 14 days allows the person time to reflect on their decision. This timeframe is shorter if the person is expected to die within 30 days.*
- *The life-ending medication is stored at a pharmacy and is delivered to the person by a registered healthcare practitioner (HCP).*
- *An HCP brings the medication, checks the person continues to retain their capacity, and a settled intention to die, and remains present.*
- *The person must administer the life-ending medication themselves.*
- *It would continue to be a criminal offence to end someone's life directly.*
- *Every assisted death would be recorded and reported for safety, monitoring, and research purposes.*

Add item

8 If the bill was to be implemented as it stands, which of the following options best characterizes your viewpoint on the proposed safeguards above?

- The safeguards are appropriate and sufficient as they stand and would not need amendments.
- The safeguards are appropriate but not sufficient as they stand and may need some amendments.
- The safeguards are not appropriate nor sufficient as they stand and need amendments/additions.
- Other

Show less

Add item

a If you selected Other, please specify your viewpoint *

Add item

Add item

b If comfortable to do so, please give the main reasons why you have made the above choice/hold the above view?

Add item

Add item

Add item

The safeguards, as shown above, were listed in the original consultation document to outline the precautions needed if the bill was to be implemented into practice.

There was criticism of these safeguards during the consultation, on the grounds that they did not go far enough to protect those in vulnerable groups, those at risk of abuse or neglect due to the actions (or lack of action) of another person e.g. young people, people living with disabilities, people living with mental health conditions etc.

Add item

9 How do you think this bill will impact vulnerable people e.g. those at risk of abuse or neglect due to the actions (or lack of action) of another person?

- It could have a positive impact on vulnerable people
- It could have no impact on vulnerable people
- It could have a negative impact on vulnerable people
- Unsure/Don't have an opinion

Show less

Add item

a If comfortable to do so, please give the main reasons why you have made the above choice/hold the above view?

Add item

Add item

p. 7 Further Comments

Add item

10 If you have any further comments on the bill, or on the topic of assisted dying in Scotland, please provide them here:

Add item

Add item

Add page

p. 8 Consent

Add item

I understand that by completing the survey and clicking finish, an indication of implied consent is taken.

All survey responses will be anonymised, so it is not possible to withdraw data after submission.

Add item

p. 9 End

Add item

Thank you for taking the time to complete the survey *Exploring perspectives of Scottish Medical Students on the proposed 'Assisted Dying for Terminally Ill Adults (Scotland)' Bill*

If there are any questions/complaints/concerns about the research, please contact either David Geddes [redacted] or Dr Jeni Harden, Usher Institute Director of Education [redacted]

Add item
